# Vitrification within a nanoliter volume: oocyte and embryo cryopreservation within a 3D photopolymerized device

**DOI:** 10.1007/s10815-022-02589-8

**Published:** 2022-08-11

**Authors:** Suliman H. Yagoub, Megan Lim, Tiffany C. Y. Tan, Darren J. X. Chow, Kishan Dholakia, Brant C. Gibson, Jeremy G. Thompson, Kylie R. Dunning

**Affiliations:** 1 Australian Research Council (ARC) Centre of Excellence for Nanoscale BioPhotonics (CNBP), Adelaide, South Australia 5000 Australia; 2 School of Biomedicine, Robinson Research Institute, University of Adelaide, Adelaide, South Australia 5005 Australia; 3 Institute for Photonics and Advanced Sensing (IPAS), University of Adelaide, Adelaide, South Australia 5000 Australia; 4 School of Physics and Astronomy, University of St Andrews, North Haugh, Scotland KY16 9SS; 5 School of Biological Sciences, The University of Adelaide, Adelaide, SA 5005 Australia; 6 Department of Physics, College of Science, Yonsei University, Seoul, 03722 South Korea; 7 School of Science, RMIT, Melbourne, VIC 3001 Australia; 8 Fertilis Pty Ltd, Adelaide, South Australia 5005 Australia

**Keywords:** Vitrification, Oocyte, Embryo, IVF, Metabolism, Photopolymerization, 3D fabrication

## Abstract

**Purpose:**

Vitrification permits long-term banking of oocytes and embryos. It is a technically challenging procedure requiring direct handling and movement of cells between potentially cytotoxic cryoprotectant solutions. Variation in adherence to timing, and ability to trace cells during the procedure, affects survival post-warming. We hypothesized that minimizing direct handling will simplify the procedure and improve traceability. To address this, we present a novel photopolymerized device that houses the sample during vitrification.

**Methods:**

The fabricated device consisted of two components: the *Pod* and *Garage*. Single mouse oocytes or embryos were housed in a Pod, with multiple Pods docked into a Garage. The suitability of the device for cryogenic application was assessed by repeated vitrification and warming cycles. Oocytes or early blastocyst-stage embryos were vitrified either using standard practice or within Pods and a Garage and compared to non-vitrified control groups. Post-warming, we assessed survival rate, oocyte developmental potential (fertilization and subsequent development) and metabolism (autofluorescence).

**Results:**

Vitrification within the device occurred within ~ 3 nL of cryoprotectant: this volume being ~ 1000-fold lower than standard vitrification. Compared to standard practice, vitrification and warming within our device showed no differences in viability, developmental competency, or metabolism for oocytes and embryos. The device housed the sample during processing, which improved traceability and minimized handling. Interestingly, vitrification-warming itself, altered oocyte and embryo metabolism.

**Conclusion:**

The Pod and Garage system minimized the volume of cryoprotectant at vitrification—by ~ 1000-fold—improved traceability and reduced direct handling of the sample. This is a major step in simplifying the procedure.

**Supplementary information:**

The online version contains supplementary material available at 10.1007/s10815-022-02589-8.

## Introduction

Cryopreservation is an essential assisted reproductive technology widely practiced in the in vitro fertilization (IVF) clinic. This technology aims to safeguard biological material for extended periods of time at cryogenic temperatures [1]. It permits the long-term banking of oocytes and embryos in liquid nitrogen (LN_2_) to ensure fertility preservation [2, 3]. Such cryopreservation permits women of reproductive age with a cancer diagnosis to preserve their fertility prior to gonadotoxic therapies [4, 5]. Additionally, it enables the preservation of gametes for social reasons or in response to a diagnosis of low ovarian reserve, thus maintaining fertility for future family expansion [6]. Furthermore, those undergoing IVF can cryopreserve surplus oocytes or embryos for future cycles without the need to undergo hormonal stimulation for oocyte retrieval [7, 8]. Finally, cryopreservation of oocytes or embryos may be utilized in donor cycles [9, 10]. Thus, oocyte and embryo cryopreservation clearly fulfill an important role for many patients. To improve the likelihood of success, cryopreservation and warming are performed by experienced embryologists due to the challenging nature of the procedure [11, 12].

A key approach in the cryopreservation of oocytes and embryos is vitrification: an ultrarapid cooling technique. Vitrification is a well-established procedure, avoiding issues of ice nucleation and crystallization by taking the sample down to the temperature of LN_2_ at a rate of ~ 10,000 to 50,000 °C/min [13–16]. Prior to vitrification, the oocyte or embryo is manually transferred into increasing concentrations of cryoprotectant [17, 18]. To ensure success, the transfer of cells must occur within a stringent time frame to minimize exposure of the sample to these cytotoxic solutions [19]. Compounding what is already a complex process, the stepwise dehydration that occurs alters cell density, leading to their rapid movement within the solution and difficulty in tracing the sample [20]. The embryologist is thus required to constantly adjust the focus of the microscope to trace the rapidly moving oocyte or embryo within the solution [17, 21]. Finally, a single oocyte or embryo is transferred within a microliter-volume (typically 1 —3 µl) onto a carrier device and directly placed into LN_2_ [15, 16, 22, 23]. The volume in which the oocyte or embryo is cryopreserved must be kept to a minimum to enable rapid cooling and avoid ice crystal formation [24, 25]. Subsequently, when a patient wishes to use their stored biological material, oocyte and embryo warming is similarly technically challenging and time-sensitive [26]. Cell revival, or warming, requires sequential exposure to solutions of decreasing concentrations of sucrose [27]. This gradually replaces the cryoprotectant, and concurrently, controls the movement of water via the osmotic gradient, thereby minimizing the speed and magnitude of swelling [28]. Procedural variation—adherence to timing and ability to trace the sample—affects cell viability following warming [29]. In summary, vitrification is a manual and labor-intensive procedure that is technically challenging [13, 30, 31]. Thus, we hypothesized that vitrification and warming would be simplified with a procedure that reduces oocyte and embryo handling and improves traceability.

We previously reported a novel device, termed the Pod and Garage, and its application for oocyte microinjection and intracytoplasmic sperm injection (ICSI) [32]. This device is comprised of a photopolymerized bespoke chamber to house the oocyte and facilitate microinjection in the absence of a holding pipette. A key attribute was that the device minimized handling and improved the traceability of injected vs non-injected oocytes, thus simplifying the procedure [32]. Our device was shown to be embryo-safe with no impact on preimplantation embryo development or DNA integrity in resultant blastocyst-stage embryos [32]. In the present study, we designed and fabricated a variant of this device and investigated its use for vitrification and warming of mouse oocytes and embryos. We determined the feasibility of the Pod and Garage for this purpose by (1) examining its suitability for cryogenic application: structural integrity following repeated immersion in LN_2_; (2) assessing oocyte viability, developmental competency and metabolism following vitrification and warming; (3) evaluating viability and metabolism of blastocyst-stage embryos following vitrification and warming; and (4) determining whether a reduction in cryoprotectant volume was compatible with vitrification and warming.

## Materials and methods

Unless otherwise stated, all chemicals were purchased from Sigma-Aldrich (St. Louis, MO, USA).

### Fabrication of the Pod and Garage

Our device is comprised of two components, the Pod and Garage, which were designed using 3D modelling software (Solidworks®, Dassault Systèmes SE, Paris, France). Fabrication of the Pods and Garages was performed using two-photon polymerization with a Nanoscribe Photonic Professional GT printer (Nanoscribe GmBH, Eggenstein-Leopoldshafen, Germany) [33] as previously described [32]. The Pods and Garages were fabricated from an IPS-photoresist resin (Nanoscribe GmBH). The dimensions were as follows: Pod (725 × 250 × 250 μm; *l* × *w* × *h*), Garage (1500 × 450 × 310 μm).

Following fabrication, the Pods and Garages were carefully removed from the glass substrate and washed as described previously [32]. Briefly, the Pods and Garages were washed three times in 5% 7X-O-Matic cleaning solution, followed by three overnight washes in filtered phosphate-buffered saline (PBS) at RT. The Pods and Garages were then stored in filtered PBS at 4 °C until use. The toxicity of the device was previously assessed using a standard mouse embryo assay (MEA) that used both negative and positive controls, with a certificate of assessment provided (IVF VET Solutions, SA, Australia) [34]. The Pods and Garage passed a standard MEA with an accepted blastocyst rate above 80% [32]. Furthermore, embryo development within the device achieved comparable, and not significantly different, embryo development rates when compared to standard culture [32].

### Animals and ethics

All experiments were approved by the University of Adelaide Animal Ethics Committee (M-2019–008) and conducted in accordance with the Australian Code of Practice for the Care and Use of Animals for Scientific Purposes.

Female (pre-pubertal, 21–23 days old) and male (6–8 weeks old) CBA × C57BL/6 first filial generation (CBAF1) mice were obtained from the University of Adelaide Laboratory Animal Services and maintained under 12 h light:12 h dark cycle with rodent chow and water provided ad libitum.

### Media

All gamete and embryo culture took place in media overlaid with paraffin oil (Merck Group, Darmstadt, Germany) at 37 °C in a humidified incubator with 5% O_2_ and 6% CO_2_ balanced with N_2_. Culture dishes were pre-equilibrated for at least 4 h prior to use. All handling procedures were performed on microscopes fitted with warming stages calibrated to maintain the media in dishes at 37 °C.

For oocyte vitrification, the base medium used for mouse ovary collection, handling, and vitrification was alpha Minimal Essential Medium (αMEM, Gibco by Life Technologies, CA, USA). Handling medium consisted of HEPES-buffered αMEM supplemented with NaHCO_3_, gentamicin sulfate, glucose, GlutaMAX (Gibco by Life Technologies, CA, USA), 5 mg/mL low fatty acid bovine serum albumin (BSA, MP Biomedicals, AlbumiNZ, Auckland, NZ), 1 mg/mL fetuin, and 20% fetal calf serum (SAFC Biosciences, Sigma-Aldrich, MO, USA). The handling medium described above constituted the base for all oocyte vitrification media.

The equilibration solution comprised of handling medium with 10% ethylene glycol and 10% dimethyl sulfoxide (DMSO). The vitrification solution comprised of 1 M sucrose dissolved in handling medium with 16.6% ethylene glycol and 16.6% DMSO. Warming solutions comprised of decreasing concentrations of sucrose (0.3 M, 0.25 M, and 0.15 M) diluted in handling medium. Vitrified oocytes were recovered in Research Fertilization medium (ART Lab Solutions, SA, Australia) supplemented with 4 mg/mL BSA for subsequent in vitro fertilization (IVF). Sperm capacitation and IVF procedures used Research Fertilization medium with 4 mg/mL BSA, and resultant embryos were cultured in Research Cleave medium (ART Lab Solutions, SA, Australia) supplemented with 4 mg/mL BSA.

For the isolation and handling of early blastocyst-stage embryos, the base medium was Research Wash medium (ART Lab Solutions, SA, Australia) supplemented with 4 mg/mL BSA. Embryos were cultured in Research Cleave medium supplemented with 4 mg/mL BSA. The vitrification, equilibration, and warming solutions used Research Wash as a base medium supplemented with 5 mg/mL BSA and prepared in the same manner as oocyte vitrification. Vitrified blastocysts were recovered in Research Cleave medium supplemented with 4 mg/mL BSA.

### Isolation of in vivo matured mouse cumulus oocyte complexes

Mice were injected intraperitoneally (i.p.) with 5 IU equine chorionic gonadotrophin (eCG; Folligon, Pacific Vet Pty Ltd., Braeside, VIC, Australia) followed by 5 IU (i.p.) human chorionic gonadotropin (hCG; Pregnyl, Merck, Kilsyth, VIC, Australia) 48 h later. At 13 h post-hCG, mice were culled by cervical dislocation and the ampullae of the oviducts dissected in warmed handling medium. The ovulated cumulus oocyte complexes (COCs) were isolated by puncturing the ampullae of oviducts with a 29-gauge × ½ inch insulin syringe with needle (Terumo Australia Pty Ltd, NSW, Australia). Cumulus cells were removed by placing COCs in hyaluronidase (500 µg/mL) diluted 1:1 in handling medium.

### Assessment of the capacity of a Pod and a Garage to withstand repeated vitrification and warming

The capacity of the fabricated Pod and Garage to withstand repeated vitrification and warming was assessed by vitrification in liquid nitrogen (LN_2_) at − 196 °C followed by instantaneous warming to 37 °C. A four-well dish (ThermoFisher Scientific, Waltham, MA, USA) was prepared with 600 µL of vitrification solutions: well 1 – research wash medium, well 2 – equilibration solution, well 3 – vitrification solution. Pods were docked into a Garage (Supp. Figure 1a) and maneuvered throughout the procedure with the aid of fine forceps. The docked Garage was placed into well 1, then transferred into well 2 and washed for 2 min 30 s (Supp. Figure 1b). The docked Garage was next placed into well 3 for 20 s and then rapidly (within 10 s) loaded onto a Cryologic Fibreplug (Cryologic Pty Ltd., VIC, Australia) (Supp. Figure 1 b). For vitrification, the Fibreplug was plunged into LN_2_. The warming process began immediately after complete immersion in LN_2_. A four-well dish containing 600 µL of decreasing concentrations of sucrose diluted in Research Wash medium was used for warming: well 1 – 0.3 M, well 2 – 0.25 M, well 3 – 0.15 M, well 4 – 0 M. The Fibreplug (containing the docked Garage) was submerged into well 1 and then the docked Garage alone, rapidly moved using fine forceps to well 2 (within 30 s), and subsequently wells 3 and 4; spending 5 min in each (Supp. Figure 1 b). The vitrification and warming procedures were repeated five consecutive times using the same set of Pods and Garage. A detailed workflow for this process can be found in Supp. Figure 1. The docked Garage was next transferred into Research Wash medium. The Pods were undocked from the Garage, and these then imaged under a brightfield microscope (Nikon SMZ1500 microscope, Nikon Instruments, Inc., NY, USA) to visualize and document any differences following the vitrification and warming procedures.

### Vitrification and warming of oocytes

Prior to COC collection, vitrification solutions were prepared in four-well dishes using 600 µL of the following: well 1 – handling medium, well 2 – 1:1 handling medium to equilibration solution, well 3 – equilibration solution, well 4 – vitrification solution. Warming dishes were prepared as described above, except for sucrose being diluted in handling medium not Research Wash medium. All dishes were warmed for 15 min at 37 °C prior to use.

Following removal of the cumulus cells, oocytes were equally divided into three groups: (1) non-vitrified which remained in handling medium on a heated stage (37 °C); hereafter referred to as *Control*, (2) vitrified using standard practice, hereafter referred to as *Standard* vitrification, and (3) vitrified within the Pods and a Garage; hereafter referred to as *Pods/Garage*. The vitrification protocol used was adapted from Zhou et al. 2016 [35]. For the *Pods/Garage* group, oocytes were loaded into Pods using a fine pulled glass pipette (one oocyte per Pod), and three Pods were then docked into one Garage (Supp. Figure 2 a). Throughout the procedure, the Pods and Garage were handled using fine forceps. The docked Garage (containing oocytes) was placed into well 1 of the vitrification dish. The docked Garage was then moved into well 2 for 2 min and washed by manually swirling the docked Garage within the solution using fine forceps (Supp. Figure 2 b). Next, the docked Garage was transferred to well 3 for 1 min 30 s and the washing procedure repeated. The docked Garage was moved into well 4 for 20 s, then quickly (within 10 s) loaded onto a Fibreplug in a minimal volume of vitrification solution and vitrified by directly plunging the Fibreplug into LN_2_. A detailed workflow for this process can be found in Supplementary Fig. 2. This procedure was performed similarly for oocytes in the *Standard* vitrification group, with the exception that oocytes were transferred between vitrification and warming solutions and directly loaded onto the Fibreplug using a micropipette and in a volume of 2 μL. The Fibreplugs (within individual sleeves) remained in LN_2_ until all oocytes were vitrified.

The warming process began immediately after all oocytes had been vitrified. Warming of the *Standard* and *Pods/Garage* groups occurred concurrently in separate four-well dishes. Fibreplugs were removed from individual sleeves and submerged into well 1 of the warming dish (Supp. Figure 2 b). Oocytes in the *Pods/Garage* group remained encased within the Pod and Garage device and transferred between warming solutions by moving the docked Garage with fine forceps. The docked Garage (containing oocytes) was quickly moved from well 1 to well 2 and subsequently to wells 3 and 4 (spending 5 min in each). Washing within each solution involved swirling of the device using fine forceps. The Pods were then undocked from the Garage and oocytes removed using a fine pulled glass pipette. Warming of oocytes within the *Standard* group occurred similarly except they were transferred between warming solutions using a micropipette and in a volume of 2 μL. Oocyte survival rate was recorded for each group. To assess developmental competency, oocytes were then transferred into pre-equilibrated 20 µL drops of Research Fertilization medium to recover in preparation for fertilization (10 oocytes/drop).

### Sperm capacitation

During oocyte recovery, a male mouse with proven fertility was culled by cervical dislocation. Sperm were isolated from the vas deferens and caudal region of the epididymis in warmed Research Wash medium then capacitated for 1 h in a pre-equilibrated dish containing a 1 mL drop of warmed Research Fertilization medium inside a humidified incubator.

### In vitro fertilization and embryo culture

Following sperm capacitation, oocytes were placed into pre-equilibrated dishes containing 90 µL drops of Research Fertilization medium at a density of 10 oocytes/drop. Oocytes were then co-cultured with 10 µL capacitated sperm for 4 h inside a humidified incubator. After fertilization had occurred, surviving embryos were transferred into fresh pre-equilibrated culture dishes containing 20 µL drops of Research Cleave medium at a density of 10 embryos/drop. Cleavage rate was recorded for each group at 24 h post-fertilization, and blastocyst rate at 96 h post-fertilization.

### Isolation and in vitro culture of mouse presumptive zygotes

Mice were injected with eCG (5 IU; i.p.), followed by hCG (5 IU; i.p.) 48 h later. Females were then paired overnight with males, and mating confirmed the following morning by the presence of a copulation plug. At 22 h post-hCG, female mice were culled via cervical dislocation and the ampullae of the oviducts dissected to isolate presumptive zygotes (PZs). Cumulus-enclosed PZs were incubated in hyaluronidase diluted 1:4 in warmed Research Wash Medium to remove cumulus cells with the aid of gentle pipetting. Following denudation, PZs were transferred into a pre-equilibrated culture dish with 20 μL drops of Research Cleave medium at a density of 10 PZs/drop. The PZs were cultured to the early blastocyst stage inside a humidified incubator for 103.5 h ± 2 h post-hCG.

### Blastocyst-stage embryo vitrification and warming

Prior to collecting blastocyst-stage embryos, vitrification solutions were prepared in four-well dishes using 600 µL of the following: well 1 and 2 – Research Wash medium, well 3 – equilibration solution, well 4 – vitrification solution. Two warming dishes were prepared in four-well dishes as described above for oocyte vitrification.

Blastocyst-stage embryos were divided into three groups: (1) non-vitrified which remained in handling medium on a heated stage (37 °C); hereafter referred to as *Control*, (2) vitrified using standard practice, hereafter referred to as *Standard* vitrification, and (3) vitrified within the Pods and a Garage; hereafter referred to as *Pods/Garage*. The vitrification protocol used was adapted from Zhou et al., 2016 [35]. For the *Pod/Garage* group, blastocysts were loaded into Pods using a fine pulled glass pipette (one embryo per Pod) and the Pods were docked into the Garage (Supp. Figure 3 a). The docked Garage (containing embryos) was placed into well 1 of the vitrification dish. The docked Garage was then transferred into well 2 and washed manually by moving the Garage with fine forceps. Next, the docked Garage was transferred into well 3 for 2 min 30 s and the washing procedure repeated. The Garage was then moved into well 4 for 20 s and quickly loaded onto the Fibreplug (within 10 s) in a minimal volume of vitrification solution and vitrified by plunging the Fibreplug directly into LN_2_. A detailed workflow for this process can be found in Supplementary Fig. 3 b. This procedure was similarly performed for embryos in the *Standard* vitrification group, with the exception that embryos were transferred between vitrification and warming solutions and loaded directly onto the Fibreplug using a micropipette and in a volume of 2 μL. The Fibreplugs (within sleeves) remained in LN_2_ until all embryos were vitrified.

After all embryos had been vitrified, the warming process began immediately as described above for the oocyte. After all groups were warmed, the Pods were undocked from the Garage and embryos removed from the Pods using a fine pulled glass pipette. Embryos were then transferred into 20 µL Research Cleave medium drops and cultured within a humidified incubator to recover prior to metabolic assessments. Blastocyst survival rate was recorded 9 h post-warming for all groups.

### Autofluorescence imaging of metabolic co-factors in oocytes and blastocyst-stage embryos

The metabolic state of oocytes and blastocyst-stage embryos was non-invasively recorded using intracellular autofluorescence and subsequent determination of the optical redox ratio (ORR). The autofluorescence intensity of flavin adenine dinucleotide (FAD), reduced nicotinamide adenine dinucleotide (NADH), and reduced nicotinamide adenine dinucleotide phosphate (NADPH) was quantified immediately after survival rate was recorded. As the spectral properties of NADH and cytosolic NADPH are very similar, separation between these molecules is difficult and are hence collectively referred to as NAD(P)H [36, 37]. Images were captured using FAD (excitation: 473 nm; emission: 490–590 nm) and NAD(P)H channels (excitation: 405 nm; emission: 420–450 nm) on an Olympus Fluoview 10i confocal microscope (Olympus Life Science, Tokyo, Japan), using a water-based 60 × objective with a final magnification of 60x, numerical aperture = 1.2 [38]. Image processing and analyses were performed using Fiji-ImageJ software (National Institute of Health, Maryland, USA). The ORR was calculated from the quantified autofluorescence intensity of FAD and NAD(P)H using the formula [39, 40]:

$$\mathrm{Optical redox ratio }(\mathrm{ORR})= \frac{\mathrm{FAD}}{\mathrm{NAD}\left(\mathrm{P}\right)\mathrm{H}+\mathrm{FAD}}$$

### Statistical analysis

All statistical analyses were performed using GraphPad Prism Version 9 for Windows (GraphPad Holdings LLC, CA, USA). The proportional data for survival, cleavage and blastocyst rates were arcsine transformed prior to statistical analysis. Results are represented as the mean ± SEM of three or more independent replicates. All experimental data were checked for normality to determine whether a parametric or non-parametric test should be used. Statistical analyses were performed using a One-way ANOVA or Kruskal–Wallis as indicated in the figure legends, and statistical significance taken at a *P*-value < 0.05.

## Results

### Design of the Pod and Garage for oocyte and embryo cryopreservation

**Fig. 1:**
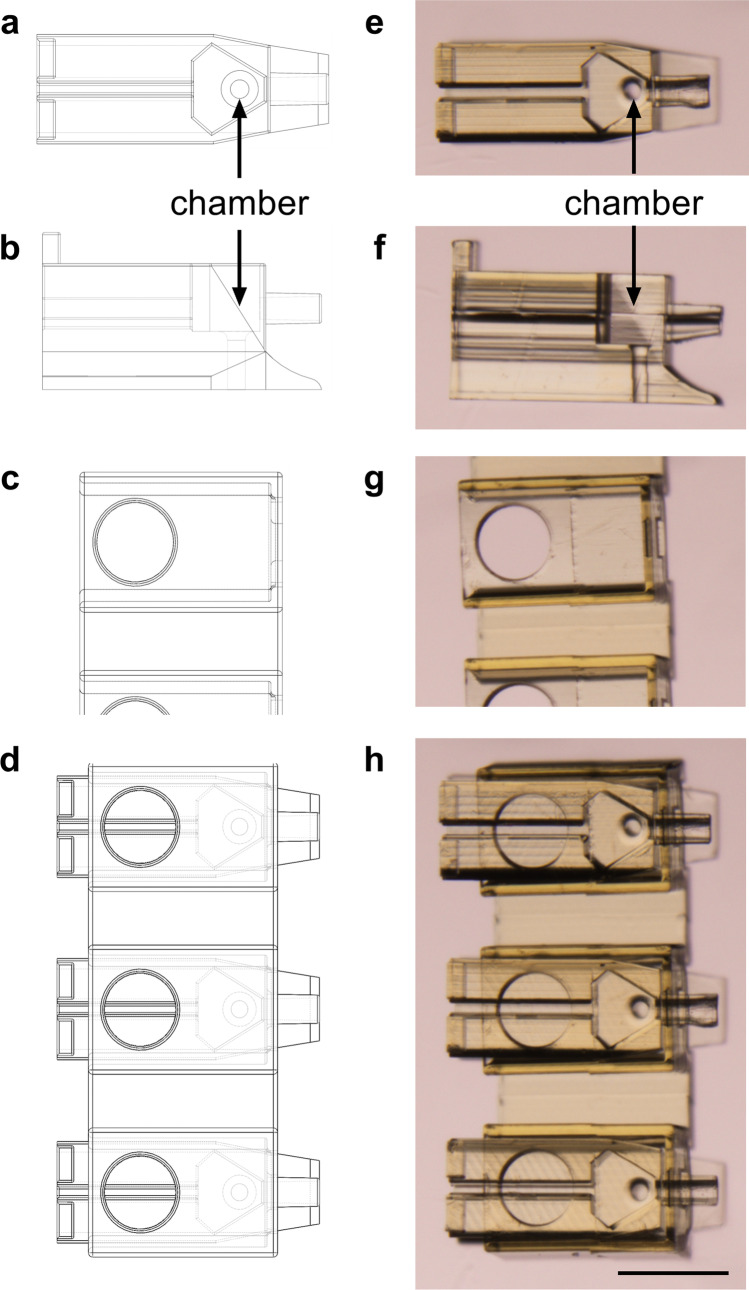
The Pod and Garage were not compromised following repeated cycles of vitrification and warming. The Pod and Garage were designed using 3D modelling software (**a**–**d**). Three-dimensional schematic of the Pod (top and side view; **a** and **b**, respectively) and Garage (top view; **c**) and an illustration of three Pods docked within a Garage (**d**) are shown. The Pod (top and side view; **e** and **f**, respectively) and Garage (top view; **g**) were fabricated using two-photon polymerization [32]. In **d** and **h**, three Pods are docked within a Garage (1500 × 450 × 310 μm; *l* × *w* × *h*). The Pod (725 × 250 × 250 μm) includes a chamber (**a**, **b** and **e**, **f**) to house a single oocyte or embryo during vitrification and warming (capacity of chamber: ~ 3 nL). Images (**e**–**h**) were taken with a final magnification of 10 × (Nikon SMZ1500 microscope, Nikon Instruments, Inc., NY, USA). Scale bar = 250 μm

The Pod (Fig. 1a, b) and Garage (Fig. 1c) were designed using 3D modelling software. Required elements of design were to (1) house the mouse oocyte or embryo; (2) seal, such that the oocyte or embryo is contained throughout processing and storage; (3) enable recovery of the oocyte or embryo following warming; (4) minimize the volume of the housing chamber and reduce the volume of cytotoxic cryoprotectant surrounding the oocyte or embryo at vitrification; and (5) enable easy handling of the device using fine forceps.

The Garage contains multiple sites into which the Pods are inserted horizontally (Fig. 1d). This device “caps” the Pod, so the oocyte or embryo cannot exit during processing or storage. Two-photon polymerization [33] was used to fabricate the Pod (725 × 250 × 250 μm: *l* ×* w* × *h*; Fig. 1e, f) and Garage (1500 × 450 × 310 μm; Fig. 1g). An image of three Pods docked within a Garage is shown in Fig. 1h. The chamber in which the oocyte or embryo is housed holds a maximum of 3 nL (Fig. 1a, b, e, f). Vitrification within 3 nL is approximately 1000-fold less than that required for standard vitrification.

### Suitability of the Pod and Garage system for cryogenic application

We first evaluated the capacity of the fabricated Pods and Garages to withstand repeated freeze-warm cycles. This was assessed by five consecutive cycles of vitrification (− 196 °C) and warming (37 °C). Following these cycles, no observable structural damage was noted (Fig. 1e–h). Furthermore, after repeated rounds vitrification and warming, the dimensions of the Pod and Garage were measured and found to be within 1% of their original dimensions.

### Oocyte survival following vitrification and warming within Pods and a Garage

**Fig. 2:**
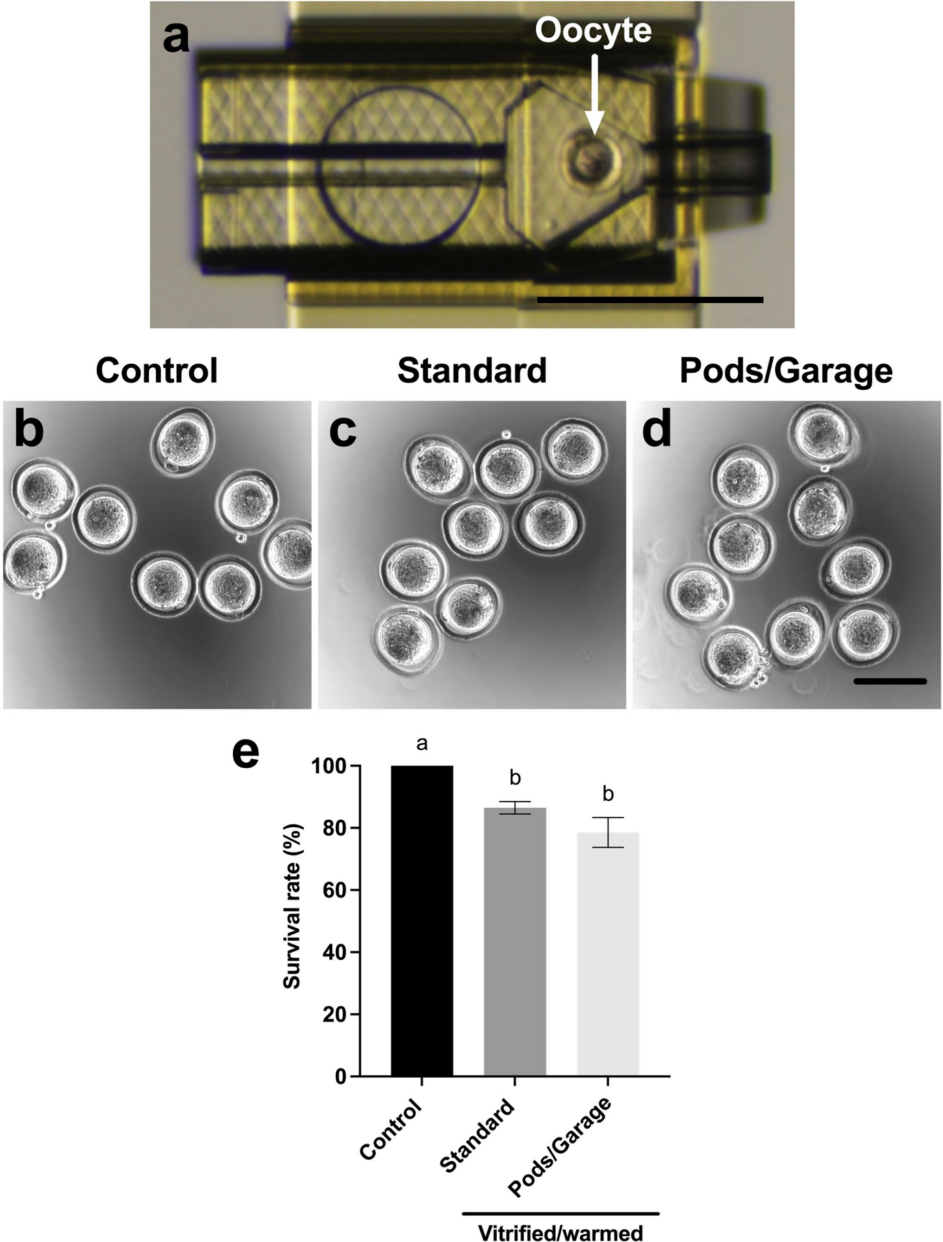
Oocytes vitrified within Pods and a Garage have a survival rate similar to those vitrified using standard practice. To assess the impact of vitrification and warming within our novel device, single oocytes were loaded into Pods (one oocyte per Pod) that were then docked into a Garage. An oocyte loaded into a single Pod docked in a Garage is shown in **a**. Oocytes were either not vitrified (*Control*; **b**) or vitrified and warmed using standard practice (*Standard*; **c**) or vitrified and warmed within Pods and a Garage (*Pods/Garage*; **d**). Oocyte survival rate was calculated for all groups following warming (**e**). Representative images were captured using a 10 × objective with a final magnification of 40 × (Nikon SMZ1500 microscope; Scale bar = 250 μm; **a**) or using a 10 × objective with a final magnification of 38 × (Olympus Fluoview 10i confocal microscope; Scale bar = 100 μm; **b**–**d**). Data in **e** is presented as mean ± SEM (n = 7 experimental replicates, representative of a total of 160–210 oocytes). Data were arcsine transformed prior to statistical testing and analyzed with a Kruskal–Wallis test, *P* < 0.05. Different superscripts denote statistical differences between groups

As we had demonstrated the suitability of our device for cryogenic application, we proceeded to assess the capability to perform oocyte vitrification and warming within Pods and a Garage. We assessed the viability of oocytes that were vitrified and warmed within our device (*Pods/Garage*) and compared this to oocytes that were either not vitrified (*Control*) or vitrified using standard practice (*Standard*). For vitrification and warming within our device, oocytes were loaded into Pods at a density of one oocyte per Pod (Fig. 2a), with three Pods docked in a Garage. Post-vitrification and warming there were no discernible differences in oocyte morphology between groups (Fig. 2b, c, d). Vitrification and warming resulted in significantly lower rates of oocyte survival in the vitrified groups (*Standard* and *Pods/Garage*) compared to those that were not vitrified (*Control*; Fig. 2e). Importantly, oocyte survival following vitrification and warming within Pods and a Garage was similar, and not significantly different from those vitrified using standard practice (*Standard*; Fig. 2e).

### Metabolic activity of oocytes vitrified and warmed within Pods and a Garage

**Fig. 3:**
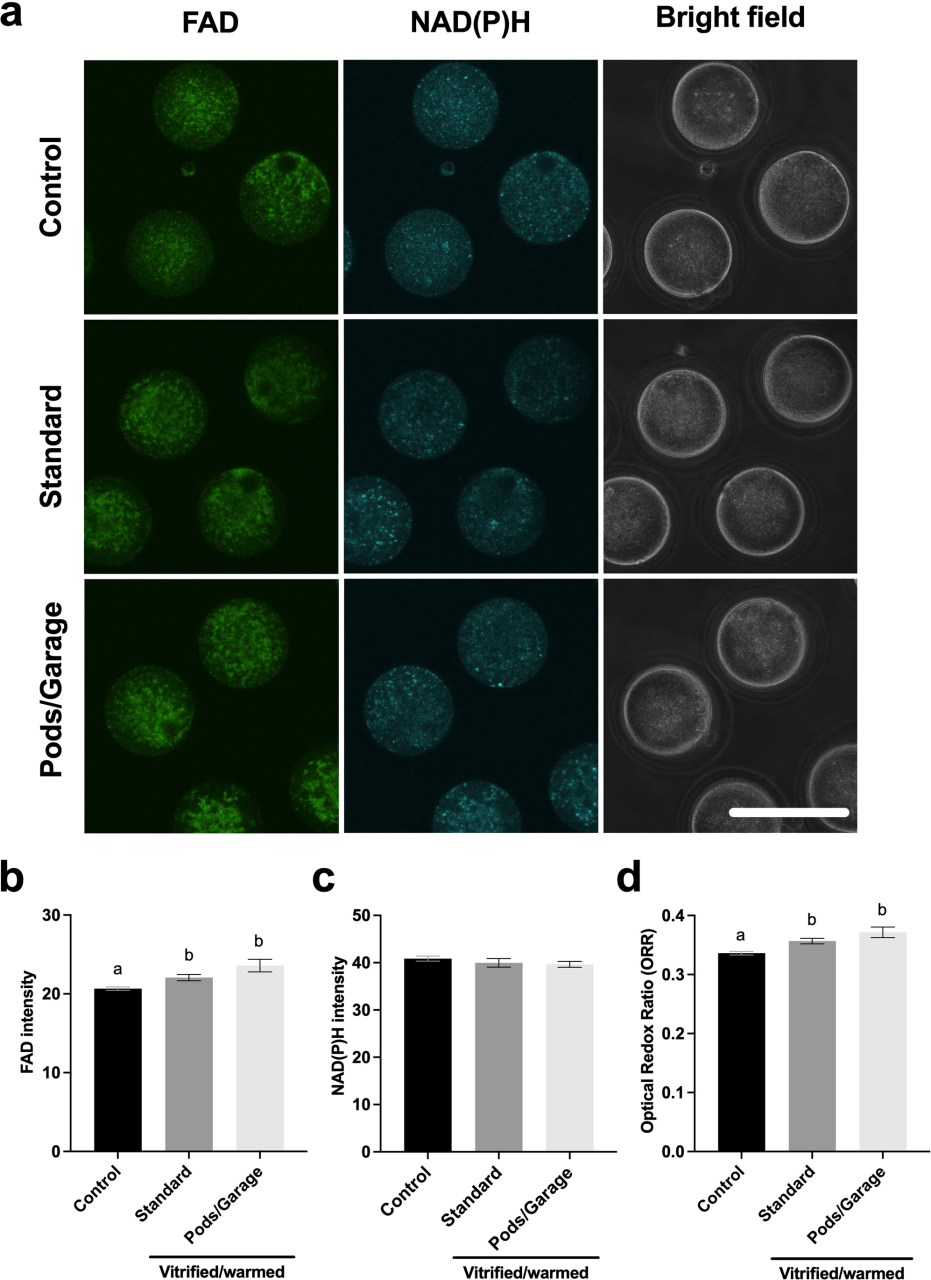
The metabolic activity of oocytes vitrified within Pods and a Garage is similar to those vitrified using standard practice. The metabolic activity of oocytes that did not undergo vitrification (*Control*), vitrified and warmed using standard practice (*Standard*) or vitrified and warmed within Pods and a Garage (*Pods/Garage*) was assessed by capturing cellular autofluorescence from the intrinsic fluorophores FAD and NAD(P)H (**a**). The relative abundance of FAD (**b**) and NAD(P)H (**c**) were quantified. The optical redox ratio (ORR; FAD/[NAD(P)H + FAD]) was also calculated (**d**). Representative images were captured using a 60 × objective with a final magnification of 60 × (Olympus Fluoview 10i confocal microscope). The first column on the left shows oocytes imaged using the FAD channel (Ex: 473 nm/Em: 490–590 nm), the middle column for NAD(P)H channel (Ex: 405 nm/Em: 420–450 nm) and the third column shows bright field images. The top, center, and bottom rows are representative images of oocytes from the *Control*, *Standard*, and *Pods/Garage* groups, respectively. All data are presented as mean ± SEM (*n* = 3 experimental replicates, representative of a total number of 27–30 oocytes). Data were analyzed using a Kruskal–Wallis test, *P* < 0.05. Scale bar = 100 μm. Different superscripts denote statistical differences between groups

We next investigated the health of vitrified and warmed oocytes by assessing their metabolic activity. We did this by recording and quantifying cellular autofluorescence from the metabolic co-factors, FAD and NAD(P)H (Fig. 3a). We found that oocytes that were not vitrified had significantly lower levels of FAD compared to the vitrified and warmed groups (*Control vs. Standard* and *Pods/Garage*; Fig. 3b). Interestingly, there was no significant difference in FAD abundance between oocytes that were vitrified using standard practice and those that were vitrified within Pods and a Garage (*Standard* vs. *Pods/Garage*; Fig. 3b). Conversely, no significant difference was found in the abundance of NAD(P)H between treatment groups (Fig. 3c). We also calculated the optical redox ratio (ORR) as an overall indicator of metabolic activity. The ORR was significantly lower in oocytes that were not vitrified (*Control*) compared to those that were vitrified (*Standard* and *Pods/Garage* groups; Fig. 3d). Importantly, there was no significant difference in the ORR between oocytes that were vitrified using standard practice or vitrified within Pods and a Garage (*Standard* vs. *Pods/Garage*; Fig. 3d).

### Developmental competence of oocytes vitrified and warmed within Pods and a Garage

**Fig. 4:**
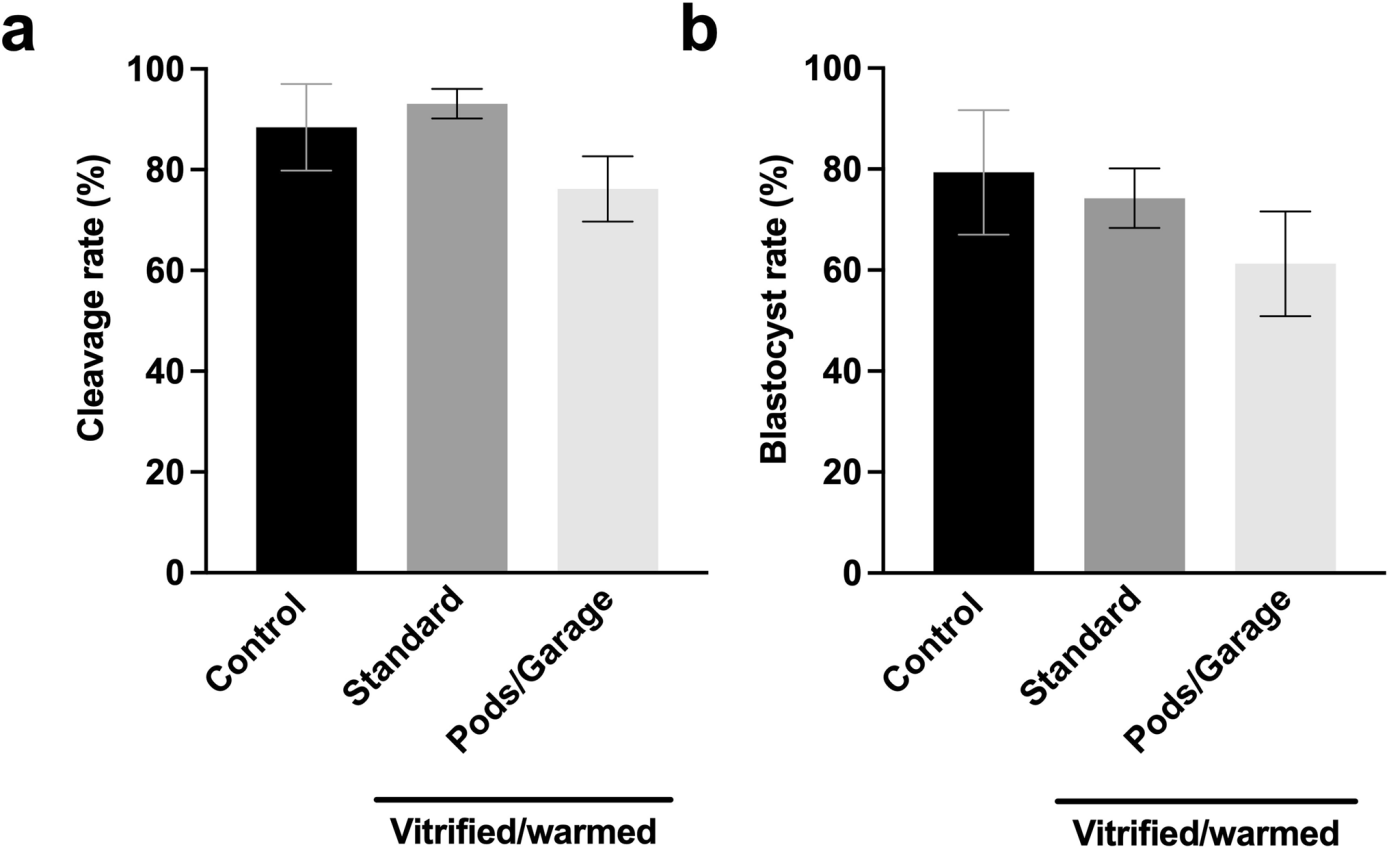
Fertilization and development to the blastocyst stage were similar between oocytes that underwent vitrification using standard practice and vitrification within Pods and a Garage. To demonstrate utility of the Pod and Garage system for oocyte vitrification, single oocytes were loaded into Pods (one oocyte per Pod) that were then docked into a Garage. Oocytes were either not vitrified (*Control*), vitrified, and warmed using standard practice (*Standard*), or vitrified and warmed within Pods and a Garage (*Pods/Garage*). Oocytes were then fertilized, and subsequent cleavage and blastocyst rates scored. Cleavage rate was calculated by the number of two-cell embryos from the starting number of fertilized oocytes (**a**). Blastocyst rate was calculated from the starting number of oocytes (**b**). All data are presented as mean ± SEM (n = 4 experimental replicates, representative of a total of 43–113 two-cell embryos and 34–101 blastocyst-stage embryos). Data were arcsine transformed prior to statistical testing and analyzed using a Kruskal–Wallis test, *P* > 0.05

We next determined whether oocyte vitrification within Pods and a Garage impacted developmental competence following warming. For this, oocytes were fertilized in vitro, and embryos cultured to the blastocyst-stage. We found no differences in fertilization (*cleavage*, Fig. 4a) or blastocyst rate (Fig. 4b) when embryos were derived from oocytes that were not vitrified (*Control*) or oocytes that underwent vitrification (*Standard* or *Pods/Garage*).

### Metabolic activity of resultant blastocyst-stage embryos following oocyte vitrification and warming within Pods and a Garage

**Fig. 5:**
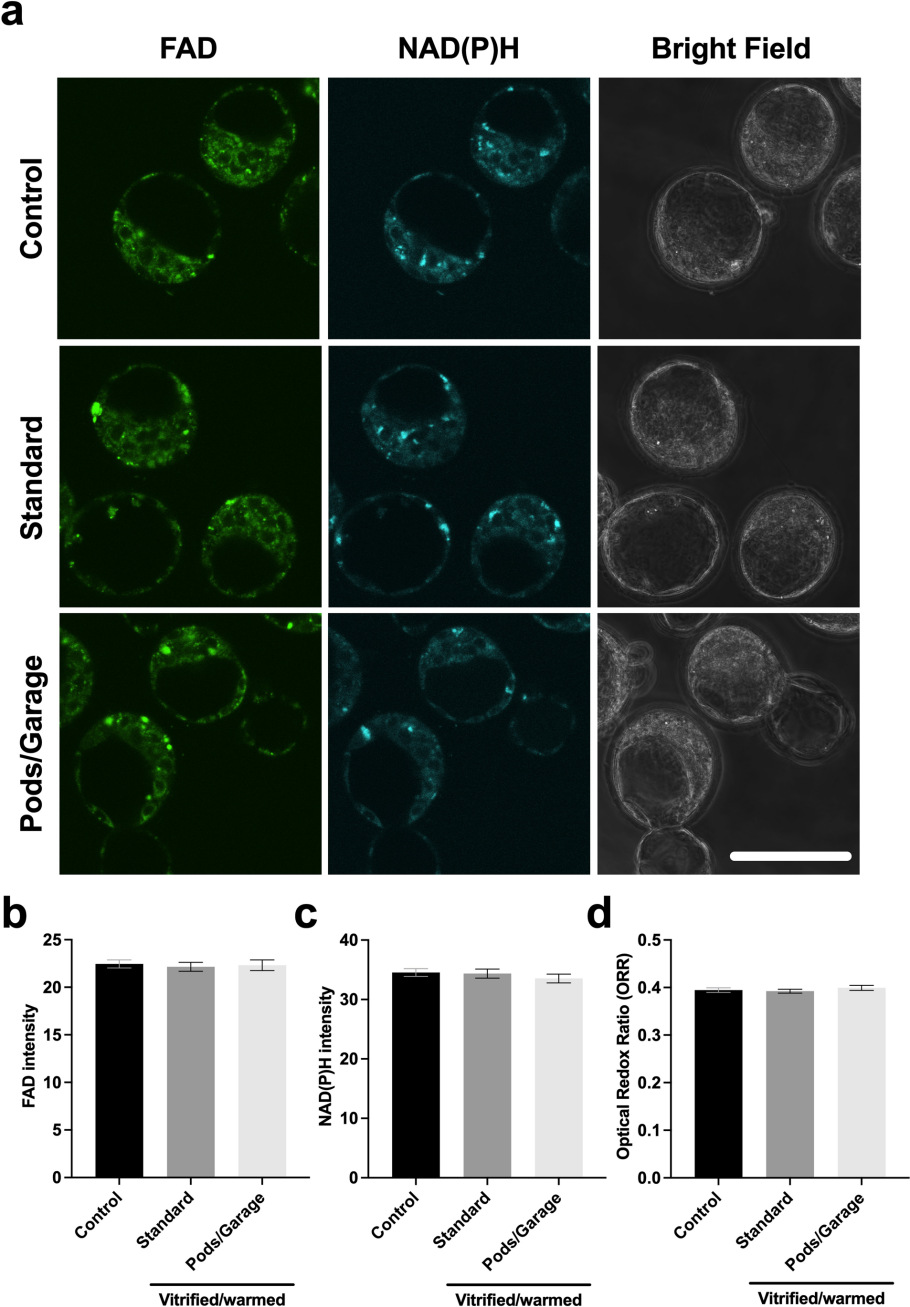
Oocyte vitrification using standard practice or vitrification within Pods and a Garage yields blastocyst-stage embryos with similar metabolic activity. The metabolic activity of blastocyst-stage embryos derived from oocytes that did not undergo vitrification (*Control*), vitrified and warmed using standard practice (*Standard*) or vitrified and warmed within Pods and a Garage (*Pods/Garage*) was assessed by capturing cellular autofluorescence from the intracellular fluorophores FAD and NAD(P)H (**a**). The relative abundance of FAD (**b**) and NAD(P)H (**c**) were quantified. The optical redox ratio was also calculated (ORR; FAD/[NAD(P)H + FAD]) (**d**). Representative images of blastocyst-stage embryos were captured using a 60 × objective with a final magnification of 60 × (Olympus Fluoview 10i confocal microscope). The first column on the left shows blastocyst-stage embryos imaged using the FAD channel (Ex: 473 nm/Em: 490–590 nm), the middle column for NAD(P)H channel (Ex: 405 nm/Em: 420–450 nm) and the third column shows bright field images. The top, center, and bottom rows are representative images of blastocyst-stage embryos from the *Control*, *Standard*, and *Pods/Garage* groups, respectively. All data are presented as mean ± SEM (*n* = 5 experimental replicates, representative of a total of 31–60 blastocyst-stage embryos). Data were analyzed using One-way ANOVA, *P* > 0.05. Scale bar = 100 μm

We also assessed the health of resultant blastocyst-stage embryos that were derived from oocytes that were not vitrified (*Control*), vitrified using standard practice (*Standard*), or vitrified within Pods and a Garage (*Pods/Garage*). We did this by assessing the metabolic activity of resultant embryos using intracellular autofluorescence from the metabolic co-factors, FAD and NAD(P)H (Fig. 5a). We found no significant differences in the abundance of FAD (Fig. 5b), NAD(P)H (Fig. 5c) or differences in the ORR (Fig. 5d) between groups.

### Early blastocyst-stage embryo survival following vitrification and warming within Pods and a Garage

**Fig. 6:**
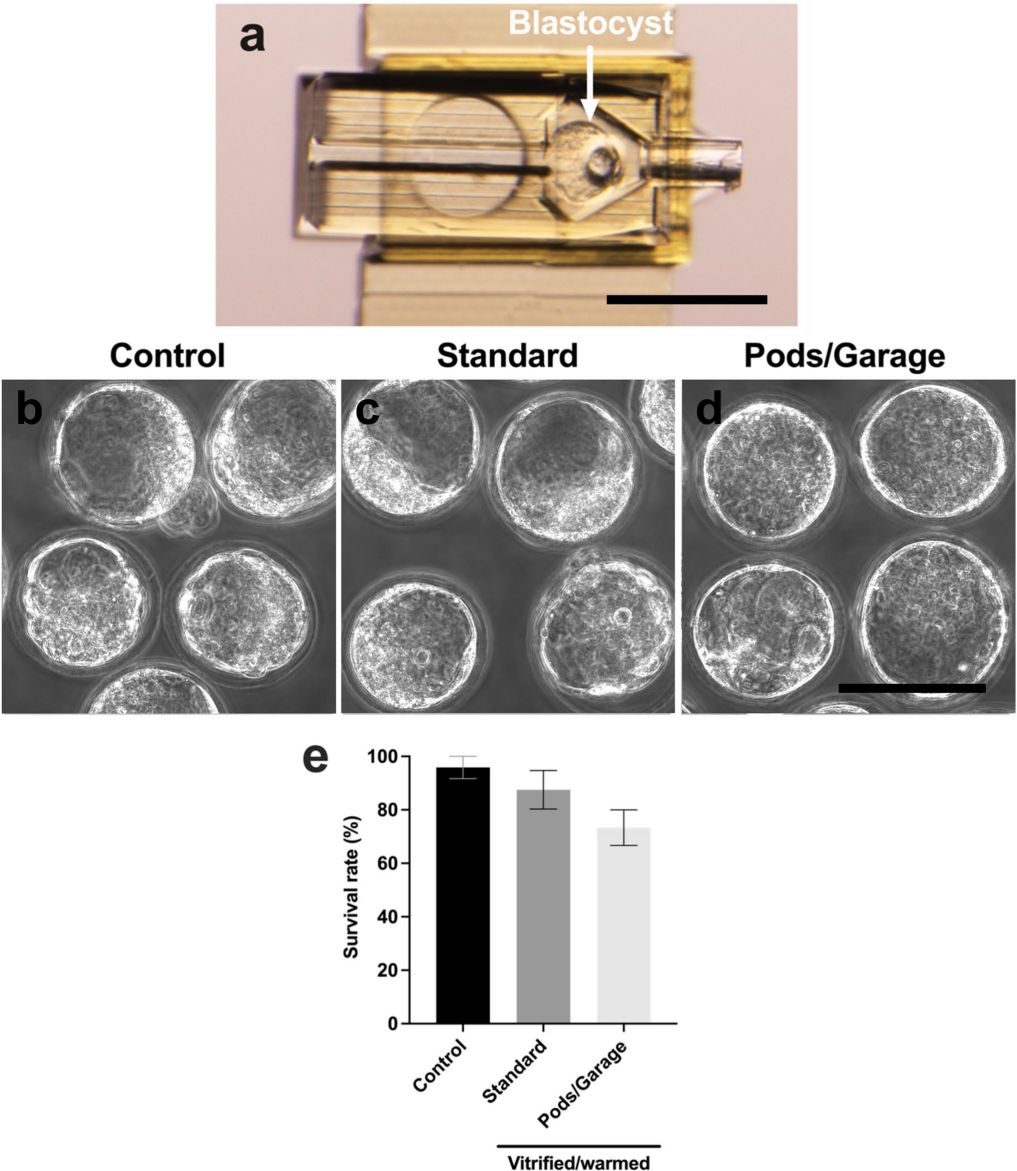
Blastocyst survival following vitrification and warming was similar between standard practice and vitrification within Pods and a Garage. To demonstrate the utility of the Pod and Garage system for embryo vitrification, early blastocyst-stage embryos were loaded into Pods (one embryo per Pod) that were then docked into a Garage. An embryo loaded into a single Pod docked in a Garage is shown in **a**. Early blastocyst-stage embryos were either not vitrified (*Control*; **b**), vitrified and warmed using standard practice (*Standard*; **c**) or vitrified and warmed within Pods and a Garage (*Pods/Garage*; **d**). Survival rate following warming was calculated for all treatment groups (**e**). Representative images were captured using a 10 × objective with a final magnification of 40 × (Nikon SMZ1500 microscope; Scale bar = 250 μm; **a**) or using a 60 × objective with a final magnification of 60 × (Olympus Fluoview 10i confocal microscope; Scale bar = 100 μm; **b**–**d**). All data are presented as mean ± SEM (*n* = 3 experimental replicates, representative of a total of 28–48 blastocyst-stage embryos). Data were arcsine transformed prior to statistical testing and analyzed using a Kruskal–Wallis test, *P* > 0.05

Following confirmation that we were able to perform vitrification and warming of oocytes within our device, we next proceeded to determine whether the Pod and Garage system was suitable for embryo vitrification. Early blastocyst-stage embryos were loaded into Pods and a Garage in a similar manner to oocytes: one embryo per Pod (Fig. 6a), with three Pods docked into a Garage. There were no observable differences in morphology between blastocyst-stage embryos that were not vitrified (*Control*); vitrified using standard practice (*Standard*); or vitrified within Pods and a Garage (*Pods/Garage*) (Fig. 6b, c, d). Encouragingly, we observed no differences in blastocyst survival between treatment groups (Fig. 6e).

### Metabolic activity of blastocyst-stage embryos following vitrification and warming within Pods and a Garage

**Fig. 7:**
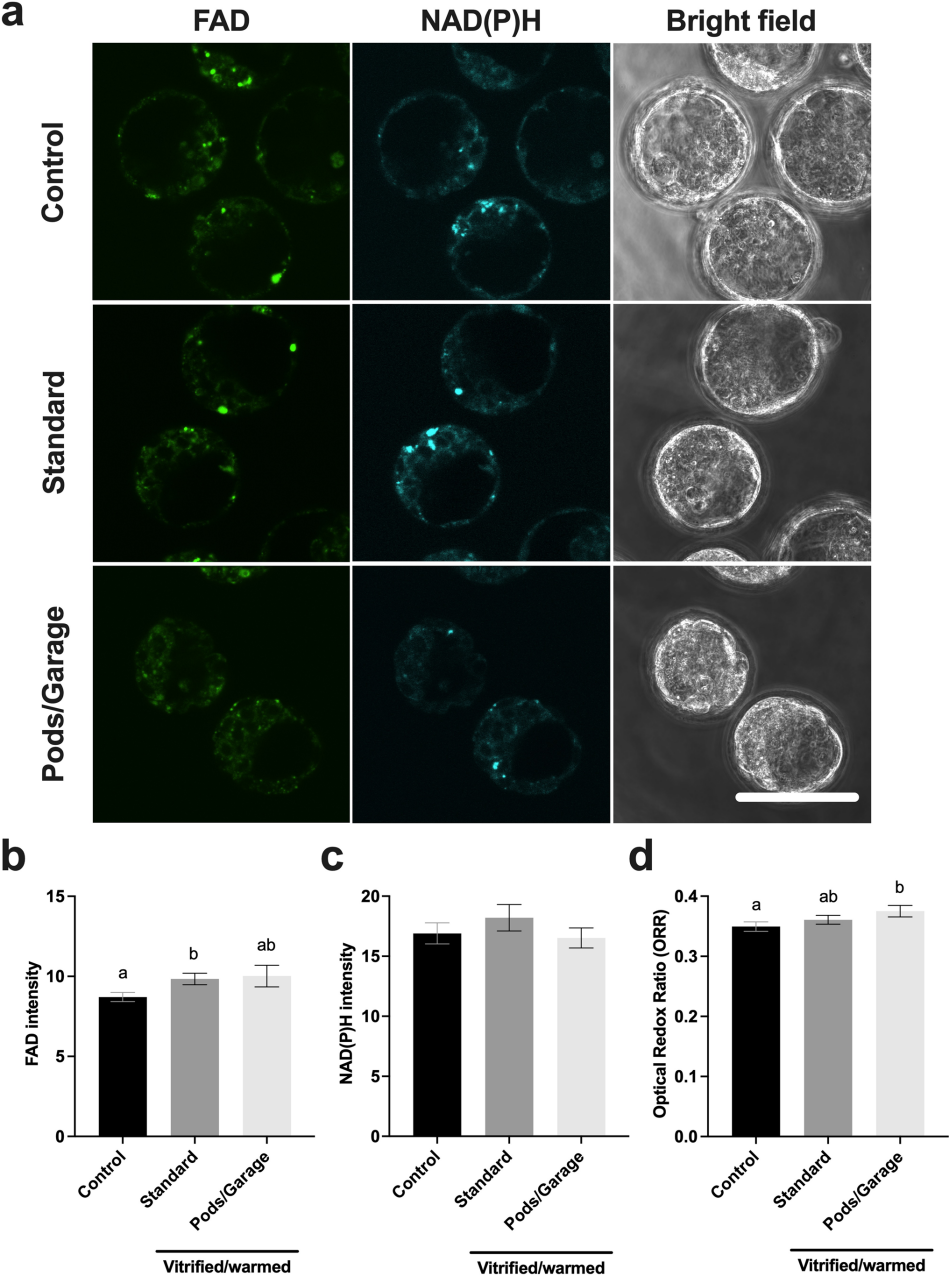
The metabolic activity of blastocyst-stage embryos vitrified within Pods and a Garage is similar to those vitrified using standard practice. The metabolic activity of early blastocyst-stage embryos that did not undergo vitrification (*Control*), vitrified and warmed using standard practice (*Standard*), or vitrified and warmed within Pods and a Garage (*Pods/Garage*) was assessed by capturing cellular autofluorescence of the intrinsic fluorophores FAD and NAD(P)H (**a**). The relative abundance of FAD (**b**) and NAD(P)H (**c**) were quantified. The optical redox ratio was also calculated (ORR; FAD/[NAD(P)H + FAD]) (**d**). Representative images were captured using a 60 × objective with a final magnification of 60 × (Olympus Fluoview 10i confocal microscope; **a**). The first column on the left shows blastocyst-stage embryos imaged using the FAD channel (Ex: 473 nm/Em: 490–590 nm), the middle column for NAD(P)H channel (Ex: 405 nm/Em: 420–450 nm) and the third column shows bright field images. The top, center, and bottom rows are representative images of blastocyst-stage embryos from the *Control*, *Standard*, and *Pods/Garage* groups, respectively. All data are presented as mean ± SEM (*n* = 3 experimental replicates, representative of a total of 28–48 blastocyst-stage embryos). Data were analyzed using the Kruskal–Wallis test, *P* < 0.05. Scale bar = 100 μm. Different superscripts denote statistical differences between groups

We next assessed the health of vitrified and non-vitrified blastocyst-stage embryos by capturing cellular autofluorescence from the metabolic co-factors, FAD and NAD(P)H (Fig. 7a). There were significantly higher levels of FAD in embryos that were vitrified using standard practice (*Standard*) compared to the non-vitrified (*Control*) group (Fig. 7b). Importantly, there was no significant difference in FAD abundance between the vitrified groups (*Standard* vs. *Pods/Garage*; Fig. 7b). For NAD(P)H, there were no significant differences between treatment groups (Fig. 7c). Interestingly, the differences in FAD and NAD(P)H resulted in a significantly higher ORR in the *Pods/Garage* group compared the *Control* group (Fig. 7d). However, there was no significant difference in the ORR between the vitrified groups (*Standard* vs. *Pods/Garage*; Fig. 7d).

## Discussion

Vitrification is a technically challenging procedure requiring rapid and precise handling of oocytes and embryos in cryoprotectants within a stringent time frame [19]. Furthermore, the process of gradual dehydration that occurs in sequential cryoprotectant solutions leads to a change in cell density and consequently, rapid, and random movement of the oocyte or embryo. This results in difficulty in the manual tracing of the precious sample. Cell survival post-vitrification and warming is highly dependent on the training and expertise of the embryologist [17]. Procedural variation during cryopreservation affects cell viability following warming [29]. Therefore, there exists a need to simplify this labor-intensive procedure and improve the traceability of oocytes and embryos. We address this in the current study using an oocyte/embryo housing device, the Pod and Garage. We previously reported application of the device for oocyte microinjection, where we demonstrated adaptability of the Pod and Garage system for intracytoplasmic sperm injection (ICSI) [32]. In the case of oocyte microinjection, our device simplified the procedure by minimizing oocyte handling and improving traceability of injected *vs.* non-injected oocytes: a difficulty that exists with standard ICSI. In the current study, the device was confirmed to simplify vitrification and warming by reducing oocyte and embryo handling and improving traceability within cryoprotectant and warming solutions.

Through rigorous testing, we demonstrated that the Pod and Garage were able to withstand repeated cycles of vitrification and warming with no obvious alterations in size or structure. This behavior is analogous to that of existing polymers used for vitrification in the IVF laboratory: their properties of low thermal conductivity, sealing performance and no requirement for external lubrication make them suitable for cryogenic application [41, 42]. To our knowledge, this study is the first to report the application of a two-photon polymerized printed device in cryopreservation.

Vitrification and warming of oocytes within the device showed no impact on viability or developmental competency when compared with the standard procedure. However, stress from handling and exposure to cytotoxic cryoprotectants negatively affected oocyte survival in both vitrification groups compared to the non-vitrified control group. A reduction in oocyte survival rate following vitrification and warming is well documented in the literature, and comparable with our results [43–45].

It is known that oocyte and embryo developmental potential is linked to their metabolism [46]. Previous studies have demonstrated that vitrification alters oocyte and embryo metabolism [47, 48]. In these studies, metabolism was measured using metabolites present in spent media. The use of label-free autofluorescence to non-invasively assess oocyte and embryo metabolism is increasing in popularity [39, 49–51]. Compared to analysis of spent medium, recording cellular autofluorescence provides a point in time measurement that also enables spatial information on the metabolic activity of individual cells [39]. The most well-studied intrinsic autofluorescent factors involved in metabolism are FAD and NAD(P)H [39, 52]. Through their use in oxidative phosphorylation, an increase in FAD abundance indicates higher metabolism, while an increase in NAD(P)H abundance is indicative of lower metabolism. A lower optical redox ratio (ORR: FAD/[NAD(P)H + FAD]) is an overall indicator of reduced cellular metabolism [40, 53]. Autofluorescence from these metabolic co-factors has been previously used to measure metabolism in oocytes and embryos [39, 40, 54]. Moreover, one study has demonstrated that vitrification of human oocytes led to altered metabolism, measured by FAD and NAD(P)H intensity [55]. However, the impact of vitrification on embryo metabolism remains unknown. Thus, we investigated the potential impact of vitrification within Pods and a Garage on oocyte and embryo metabolism by quantifying the intensity of these endogenous fluorophores.

We found the metabolic activity of oocytes was similar in the vitrification groups (*Standard vs*. *Pods/Garage*), indicating that vitrification and warming within our device resulted in oocytes with similar metabolic health to those vitrified using standard practice. Interestingly, the process of vitrification and warming itself altered the metabolism of oocytes compared to those that were not vitrified. An increase in metabolism in the vitrified groups may be necessary for cellular recovery post-warming. However, it is worth noting that this effect on metabolism was lost in resultant blastocysts developed from vitrified oocytes. Additionally, our device supported vitrification and warming of early blastocyst-stage embryos with no impact on survival or variation in metabolic activity compared to those that were vitrified using standard procedure. As with oocytes, the differences in metabolism between the vitrified groups and the non-vitrified control group observed may indicate an increased demand for energy required for post-warming recovery processes [47]. Hence, this study highlights the need to consider the impact of cryopreservation on metabolism when developing diagnostic tools that use cellular autofluorescence to predict developmental competency [39, 51, 56, 57].

Under standard vitrification, the oocyte or embryo is sequentially transferred into increasing concentrations of cryoprotectant using a micropipette [58]. An embryologist manually handles an individual oocyte or embryo during the procedure [17]. As the oocyte or embryo is passed through these cryoprotectant solutions, dehydration occurs resulting in random and rapid movement of the sample. Consequently, tracing the position of the oocyte or embryo is often difficult, and the sample can even be lost during the procedure [59]. Furthermore, difficulty in tracing the sample can lead to increased time spent in the cytotoxic cryoprotectant which is known to negatively impact oocyte and embryo viability [22]. Conversely, our device houses a single oocyte or embryo within a Pod with multiple Pods docked within a Garage. This serves two purposes: 1) the processing of multiple oocytes or embryos at a time and 2) the Garage “caps” the Pod to ensure the sample is not lost during processing. Thus, our novel device overcomes the issues of traceability, handling, and low throughput. The Pod and Garage brings a critical step-change to the vitrification procedure.

One crucial point during vitrification is the volume of cryoprotectant in which the oocyte or embryo is vitrified [60]. It is essential that the volume of cryoprotectant is minimized to achieve a higher cooling rate which in turn, avoids ice crystal formation [24]. In standard practice, oocytes and embryos are typically vitrified in 1–3 μL of cryoprotectant [16], with some devices enabling the control of drop sizes down to a volume of ~ 0.1 μL [61, 62]. In contrast, within our device, the vitrification solution was dramatically lower as the chamber in which a single oocyte or embryo is housed was approximately 3 nL. This demonstrates that vitrification within our system can be performed within a nanoliter volume: an approximate 1000-fold reduction in the volume of toxic cryoprotectant solution compared to standard practice. Given the small volume contained within a Pod, our current method of manually moving the device from one cryoprotectant solution to another relies on passive movement of solutions. To improve on this, future studies will investigate whether utilization of a microfluidic pump to actively replace vitrification and warming solutions within our device improves survival and developmental potential. Furthermore, prior to implementation in the IVF clinic, embryo viability should be further investigated by examining implantation rate and fetal health following embryo transfer in other mammalian models.

In conclusion, vitrification and warming within our device simplified the procedure by minimizing the direct handling of oocytes and embryos as well as improving traceability. Overall, the presented Pod and Garage system was successfully used in vitrification and warming of oocytes and embryos and provides a platform to advance the field of cryopreservation.

## Supplementary information

Below is the link to the electronic supplementary material.

Supplementary file1 (DOCX 4777 KB)

## Data Availability

All data generated or analyzed during this study are included in this published article and are available from the corresponding author on reasonable request.

## Code availability

N/A.
