# Correction to: Vitrification within a nanoliter volume: oocyte and embryo cryopreservation within a 3D photopolymerized device

**DOI:** 10.1007/s10815-022-02610-0

**Published:** 2022-09-07

**Authors:** Suliman H. Yagoub, Megan Lim, Tiffany C. Y. Tan, Darren J. X. Chow, Kishan Dholakia, Brant C. Gibson, Jeremy G. Thompson, Kylie R. Dunning

**Affiliations:** 1 Australian Research Council (ARC) Centre of Excellence for Nanoscale BioPhotonics (CNBP), Adelaide, South Australia 5000 Australia; 2 School of Biomedicine, Robinson Research Institute, University of Adelaide, Adelaide, South Australia 5005 Australia; 3 Institute for Photonics and Advanced Sensing (IPAS), University of Adelaide, Adelaide, South Australia 5000 Australia; 4 School of Physics and Astronomy, University of St Andrews, North Haugh, St Andrews, Scotland KY16 9SS; 5 School of Biological Sciences, The University of Adelaide, Adelaide, SA 5005 Australia; 6 Department of Physics, College of Science, Yonsei University, Seoul, 03722 South Korea; 7 School of Science, RMIT, Melbourne, VIC 3001 Australia; 8 Fertilis Pty Ltd, Adelaide, South Australia 5005 Australia

**Correction to: Journal of Assisted Reproduction and Genetics**

**https://doi.org/10.1007/s10815-022-02589-8**

The correct wavelength of the laser used to excite an intracellular fluorophore should be “405 nm” and not “305 nm”.

The original article has been corrected.

