# Supplementary material for: Vitrification within a nanoliter volume: oocyte and embryo cryopreservation within a 3D photopolymerized device
Source: J Assist Reprod Genet. 2022 Aug 11;39(9):1997–2014. doi: 10.1007/s10815-022-02589-8 (PMC9474789; doi:10.1007/s10815-022-02589-8)

**Supplementary Figure 1. Workflow used to assess the ability of the device to withstand repeated vitrification and warming.** Throughout the procedure the device was handled using fine forceps. Three empty Pods were docked into a Garage (**a**) and moved through vitrification and warming solutions (**b**). For vitrification, the device was loaded onto a Fibreplug and the Fibreplug plunged into liquid nitrogen (**b**) Pods were undocked from the Garage and integrity assessed. (*RW*: Research Wash; *ES*: equilibration solution; and *VS*: vitrification solution).


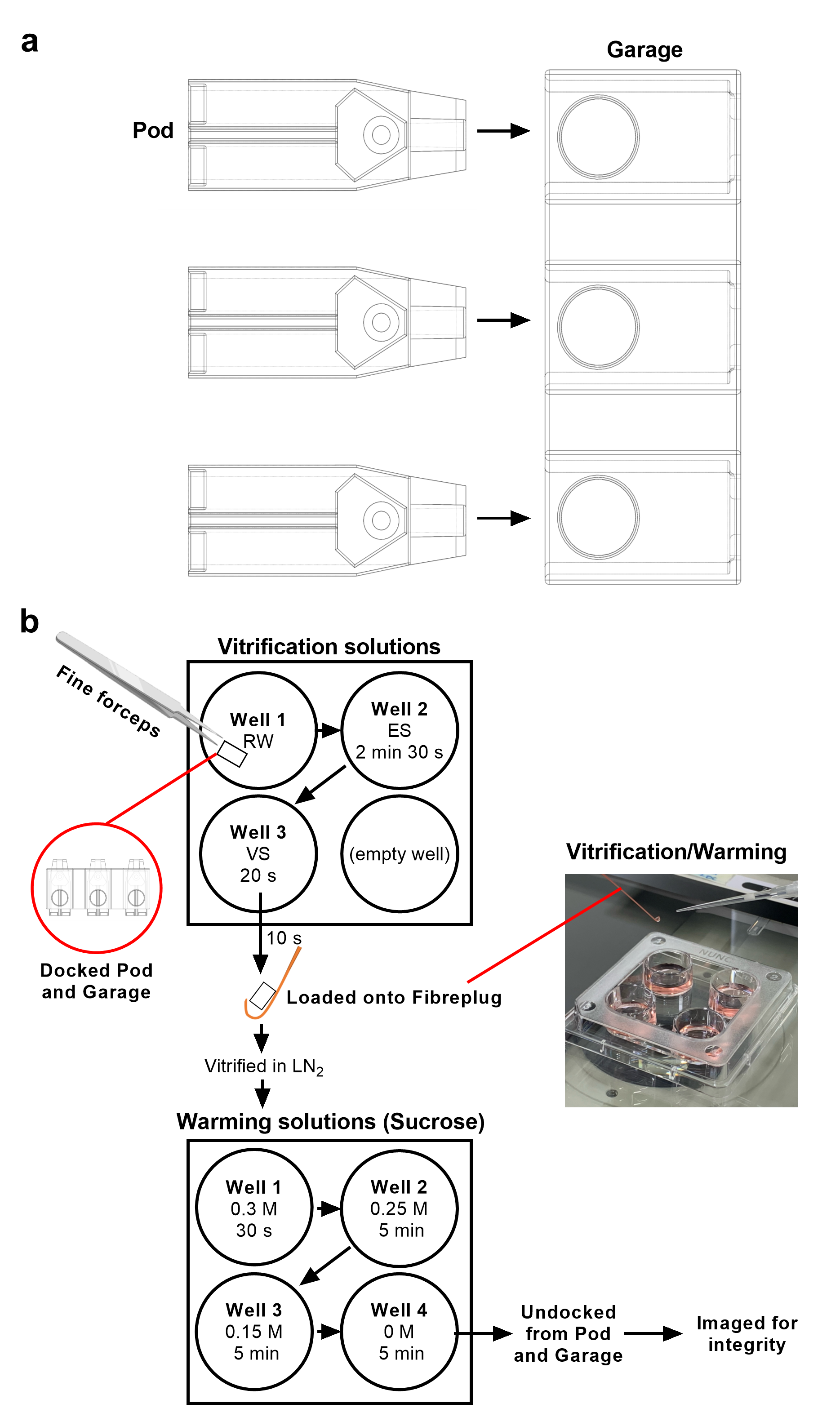


**Supplementary Figure 2. Workflow: oocyte vitrification and warming within Pods/Garage.** The device was handled using fine forceps. Oocytes were individually loaded into Pods using a fine glass pipette and the Pods then docked into a Garage (**a**). The device was moved through vitrification and warming solutions. For vitrification, the device was loaded onto a Fibreplug and the Fibreplug plunged into liquid nitrogen (**b**). Pods were undocked from the Garage. Oocytes were removed and underwent viability assessments (**b**) (*HM*: handling medium; *HM:ES*: 1:1 dilution of HM:equilibration solution; *ES*: equilibration solution; *VS*: vitrification solution).


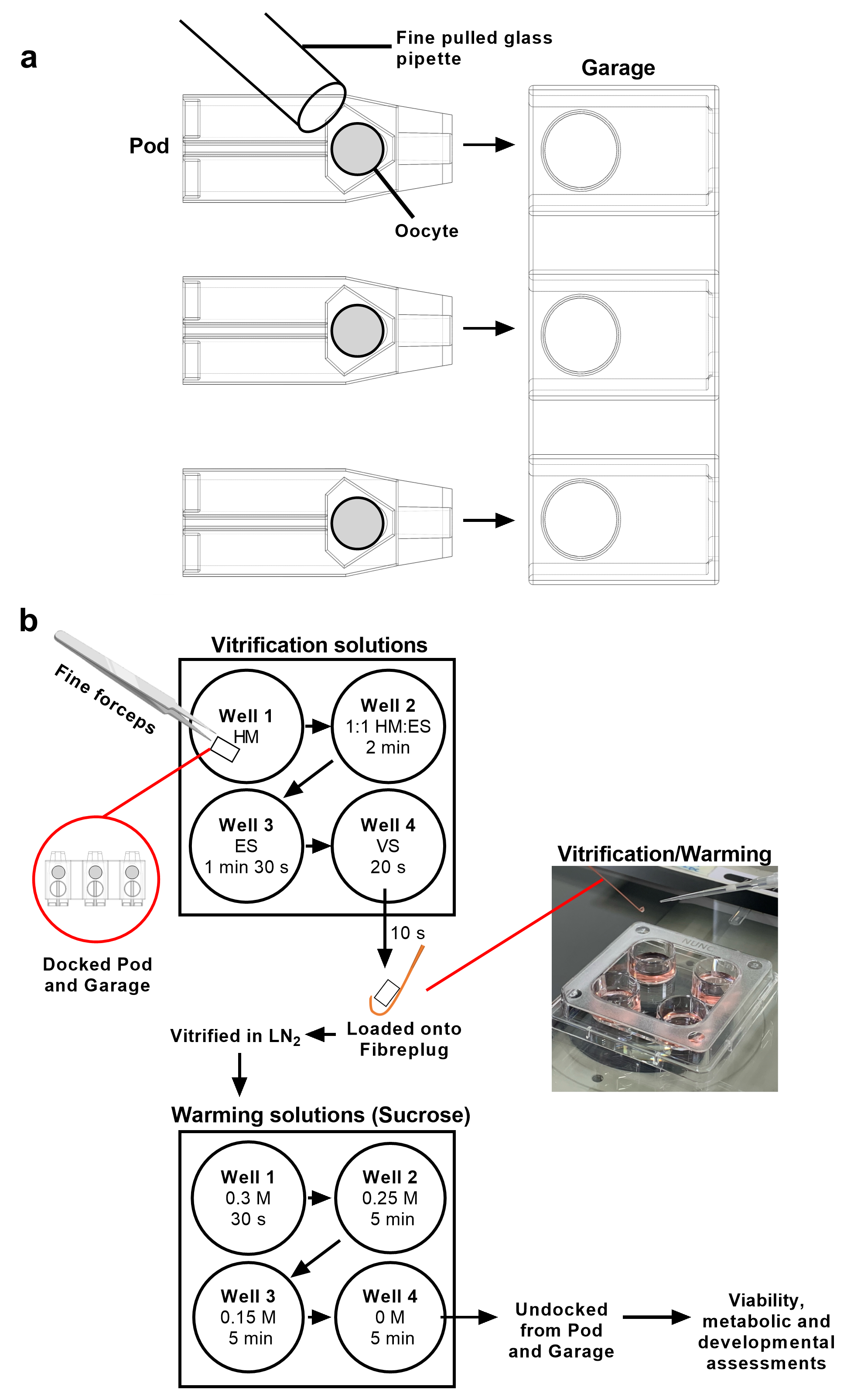


S**upplementary Figure 3. Workflow: blastocyst vitrification and warming within Pods/Garage.** The device was handled using fine forceps. Embryos were individually loaded into Pods using a fine glass pipette and the Pods then docked into a Garage (**a**). The device was moved through vitrification and warming solutions. For vitrification, the device was loaded onto a Fibreplug and the Fibreplug plunged into liquid nitrogen (**b**). Pods were undocked from the Garage. Embryos were removed and underwent viability assessments (**b**) (*RW*: Research Wash; *ES*: equilibration solution; and *VS*: vitrification solution).


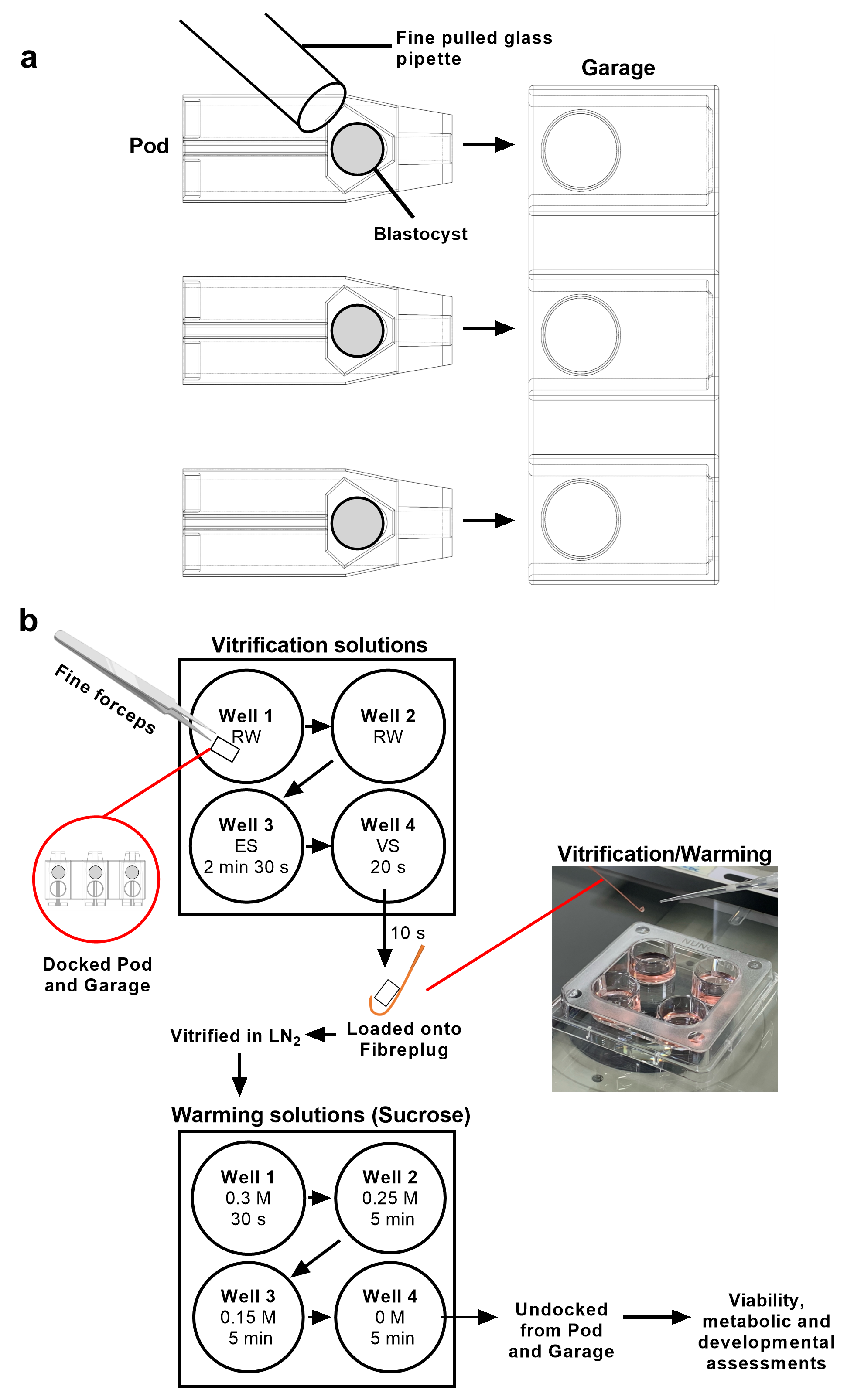

Supplement: Supplementary file 1 — Supplementary file1 (DOCX 4777 KB) [file 10815_2022_2589_MOESM1_ESM.docx]
